# A Mixed-Methods Study to Develop a Resilience Scale for Thai Elderly with Chronic Diseases and Depression

**DOI:** 10.1155/2022/3256981

**Published:** 2022-01-15

**Authors:** Kanokporn Thongkhum, Narisara Peungposop, Nanchatsan Sakunpong

**Affiliations:** Behavioral Science Research Institute, Srinakharinwirot University, Thailand

## Abstract

This study was an exploratory sequential mixed-methods design to develop a resilience scale for Thai elderly with chronic diseases and depression. The qualitative findings from the focus group discussion with 6 participants were used to develop a resilience scale, and the scale was then tested on 310 samples to check the reliability and validity of the scale. The qualitative results showed that resilience was defined in 3 themes: My Characteristics, My Abilities, and My Dependencies, which were composed of 9 different categories. The results of the quantitative examination showed that all 21 items of the resilience scale had a good corrected item-total correlation and the Cronbach's alpha coefficient of 0.85 indicated that the scale was internally consistent and highly reliable. The construct validity of the resilience scale was tested by confirmatory factor analysis and revealed that the resilience model was consistent with the empirical data based on the goodness-of-fit index (chi − square = 161.51, df = 186, *p* value = 0.90, RMSEA = 0.000). All the results show that the resilience scale has excellent and appropriate psychological properties. Health-care workers can use the resilience scale to assess the elderly and develop a resilience-promoting program specifically for the elderly with chronic diseases and depression to improve the well-being of the elderly.

## 1. Introduction

The rapid increase in the aging population worldwide has an impact on the economic and social situation of the country [1]. In Thailand, the situation of the elderly has also continued to increase. Therefore, the elderly is a large population group that needs to be given attention to in terms of the development and promotion of a good quality of life [2]. This is because the elderly has biological changes that affect mental functions and interpersonal relationships [1]. This affects the health and well-being of elderly people [3], reduces life satisfaction [1], and leads to depression and suicidal thoughts [2, 4]. It has been found that many elderly people die from chronic diseases every year. This is because chronic or noncommunicable diseases (NCDs) are a group of diseases that have a long duration of illness. They lead to illness, disability, loss of mental health, decreased quality of life, and many premature deaths [5, 6]. In addition, depression has been found to be an increasingly common mental health problem in older adults and one of the most common causes of chronic disease burden [6–8]. Therefore, it is important to promote and develop mental health in addition to physical health care for elderly people with chronic diseases and depression [2, 3]. In order for elderly people to be able to face life's adversities and bounce back to a normal daily life [1]. A review of the literature found that high resilience can reduce depression and improve elderly peoples' perceptions of mental and physical functioning. As a result, the elderly had a better quality of life [9].

Resilience is a person's ability to prevent or overcome the effects of adversity. It is the development of a person's flexibility and strength to move forward even in the face of adversity. Resilience is composed of three factors: the external resources available to a person (I have), the person's internal strengths (I am), and the interpersonal and social skills the person possesses (I can). A highly resilient older person is able to cope, adapt, and respond positively to crises events and can bounce back to a normal life [10]. Therefore, elderly people with chronic disease and depression should continuously develop their resilience to cope with and effectively overcome crises in their lives. A review of the literature on resilience found several studies on resilience in older adults [10, 11] and studies on resilience in older adults with various chronic diseases [12, 13] or resilience in elderly people with depression [14, 15], which was a study in disease-specific samples. In addition, studies of resilience scale development were also found in older adults who did not have a disease [16–18]. Studies of resilience scale development in elderly people with chronic diseases and depression have not yet been found to assess and develop resilience in this group.

This study is aimed at investigating the attributes of resilience in elderly people with chronic diseases and depression. A qualitative research method was used to obtain the essential information from real events [19] and use the qualitative results to develop a resilience scale. Then, a quantitative test was conducted to verify the psychometric properties. This resilience scale could be used to assess and develop a resilience program specifically for elderly people with chronic diseases and depression. This will be useful for promoting the well-being of elderly people in the future [17].

## 2. Materials and Methods

This study was a sequential exploratory design with mixed methods [20]. It consisted of two phases. The first consisted of qualitative research to investigate the characteristics of resilience in older people with chronic illness and depression, and the second phase consisted of quantitative research to establish and develop the validity and reliability of the resilience scale. This study was approved by the Human Research Ethics Committee at Srinakharinwirot University, Thailand.

### 2.1. Qualitative Phase: Phase I

The researchers collected data through focus group discussions (FGDs) because group discussions revealed a wide range of sensitive personal information and some sensitive topics emerged only in the context of group discussions [21]. There have been several studies that used FGDs in the exploratory phase of mixed methods to design the scale and investigate the psychometric properties [22–25].

#### 2.1.1. Setting and Participants

This study was conducted in rural areas of Thailand. The majority of the population were farmers and general employees. The six participants (3 males, 3 females) in the FGDs met the inclusion criteria: 60-69 years old, primary education, no dementia (MMSE-Thai 2002 >17 points): assessed by the Mini-Mental Status Examination-Thai 2002 [26], diabetes and/or hypertension, and cured depression. Scores on the 9-question rating scale for depression (9Q) were less than 7 points (no depression) [27] and scores on the resilience quotient (RQ-20) were greater than 69 points (resilience above normal) [28]. These scores were the positive results indicating a high level of resilience in the face of critical events [29]. The researchers selected a purposive sample to conduct the FGDs because we wanted to use the results of the data analysis to create the Resilience Scale for the elderly with chronic diseases and depression. The key informants must be the elderly with chronic diseases who had cured depression in the past and had high resilience scores. These indicated that they were able to cope with the adversities of depression [9] and were the sample groups that provided rich information according to the objective of this study.

#### 2.1.2. Method

The researchers established relationships with the 6 participants prior to the 2-week group discussion. This was followed by three 2-hour sessions in a quiet room where they were not disturbed. Information from these sessions was recorded using two tape recorders, as were the researcher's notes. The questions used in the group discussions were the following: (1) what thoughts, feelings, or emotions did you use to overcome adversity? (2) How did you continue to live after facing adversity? (3) How did other people or certain things help you overcome adversity? The researchers then transcribed the group discussions from the audio recorder and organized this data along with the raw data from the recordings and field observations.

#### 2.1.3. Data Analysis

The researchers analysed the data inductively according to the research questions and assessed the quality of the qualitative research based on trustworthiness [30] through the following processes: (1) credibility: the researchers worked in the same field as the field research. Therefore, they were confident that the participants could be trusted to cooperate in providing information. After coding the data, categorizing the codes, and determining the themes, the researchers used the technique of member checking by returning the results to the participants to verify the correct interpretation of the data. Triangulation involved comparing the data from the FGDs with the coded data and categorized codes three times and cross-checking the results by two consultants to ensure that the facts were credible. (2) Transferability: the researchers interpreted the participants' information and its context in detail and clearly so that other researchers could use the information to their advantage. (3) Reliability: researchers examined peer reviewers' traces of findings in qualitative research and examined the links between research questions, research data, and research findings. (4) Confirmability: the findings of the qualitative research were openly truthful and were confirmed by the consultant's examination of the traces of the research process.

### 2.2. Quantitative Phase

The researchers used quantitative research methods to assess the content validity and reliability of the resilience scale, which had a 5-point Likert scale [31] resulting from the synthesis of operational definitions and questionnaire development based on the results of the qualitative study.

#### 2.2.1. Setting and Participants

The sample consisted of elderly people with chronic diseases and depression. They were aged between 60 and 69 years and lived in rural areas of Thailand. Most of them were employees and farmers, had completed primary school, did not suffer from dementia (MMSE-Thai 2002 >17 points), had diabetes and/or hypertension, and were under treatment for depression. They had mild to moderate depression (9Q = 7 − 18) and lower than normal resilience (RQ < 55), suggesting that the elderly were unable to cope with crisis events. The researchers selected a purposive sample of 340 individuals in total, representing an older population with chronic illness and depression. Of these, 30 samples were used for the test of item discrimination and reliability and 310 samples were used for the test of construct validity to measure the psychometric properties of the resilience scale developed by the researchers.

#### 2.2.2. Method

The researchers conducted the study in the following steps: (1) synthesizing operational definitions based on qualitative research findings and construct questions; (2) testing the quality of the preliminary measurement; and (3) testing structural validity through confirmatory factor analysis. The researchers collected data from elderly people with chronic diseases and depression who were patients in 6 health-care facilities in the lower central provinces of Thailand and met the inclusion criteria. The resilience scale was tried out with a sample of 30 individuals who had similar characteristics to those used for the quantitative test. The researchers tested the created resilience scale with a sample of 310 individuals. They then checked the completeness of all measurements to analyze the data and further test the hypothesis.

#### 2.2.3. Data Analysis

**Figure 1:**
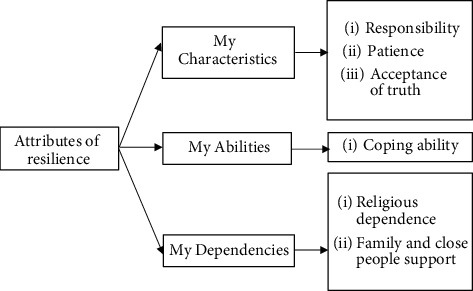
The components of resilience.

The researchers synthesized the operational definition to define resilience in elderly people with chronic diseases and depression. Which was the adaptability of older people and their ability to bounce back after a crisis. It relates to behaviors, thoughts, and actions of elderly people that can learn and develop. The core components of resilience had 3 domains and consisted of 9 subcomponents (Figure 1). The researchers used the components of resilience to develop 39 questions. The researchers then checked the quality of the preliminary scale and tested the construct validity as follows.

*(1) Content Validity*. The content validity of the draught 39-item resilience scale was examined by three experts in the fields of psychology, behavioral sciences, and qualitative research to consider item operational definition (IOC) congruence by considering the selection of items with an IOC value of 0.50 or higher [32]. A total of 26 qualifying items were identified. The researchers examined the quality of the 26-item resilience scale by trying it out on a sample of 30 elderly people with chronic diseases and depression who were similar to the research sample group.

*(2) Item Discrimination*. The researchers assessed the discriminative power of the 26-item resilience scale by analyzing the total correlation of the items. The items with a corrected item-total correlation (CITC) of less than 0.20 were eliminated [33]. A total of 21 qualifying items were found.

*(3) Reliability*. The researchers tested the reliability of the 21-item resilience scale (3 components of resilience) by calculating Cronbach's alpha coefficient [34] to check the internal consistency of the scale. Here, the generally acceptable Cronbach's alpha must be greater than 0.70.

*(4) Construct Validity*. The researchers examined the structural validity of the resilience scale constructed with 21 items, which was the observed variable in the three components of resilience. The resilience scale was tested on a sample of 310 elderly people with chronic diseases and depression. Subsequently, the scale was tested for construct validity by confirmatory factor analysis (CFA) [35] using the LISREL version 8.72 program to determine whether the theoretical hypothesis of the resilience model was consistent with the empirical data.

## 3. Results

### 3.1. Qualitative Phase

The qualitative results of the FGDs showed that the attributes of resilience in elderly people with chronic diseases and depression were defined in 3 themes: (1) My Characteristics, (2) My Abilities, and (3) My Dependencies. These comprised 9 categories, as shown in the square frame (Figure 1).

Theme 1: My Characteristics: an inner quality of a person that is able to recover after a crisis. It is a feeling, attitude, and beliefs that are expressed until they become a habit. It consists of 5 categories: Responsibility, Patience, Acceptance of Truth, Self-Confidence, and Self-Satisfaction.

*“I have adopted two grandchildren and I have to take care of them as best I can.”* (Aunt Jib).

*“I took care of my wife since she was sick until she died. It was normal to regret that. I thought it was her time. I had to accept it.”* (Uncle Kat).

*“I was able to live by my own and earn my own living without depending on anyone.”* (Uncle Ka).

Theme 2: My Abilities: a person's internal ability to maintain interpersonal and social skills during a crisis. The only category was coping skills.

*“My wife ran off with the money we had saved together. I try not to get angry, then I come back to consciousness and think what is the reason for this incident. Then I thought to myself that I should not remember this and continue to earn my money.”* (Uncle Ka).

Theme 3: My Dependencies: a person's perception of receiving support from other people or other sources of social benefits. It consisted of 3 categories: religious dependence, support from family and close people, and financial dependence.

*“After my wife died, I had relatives and children who always comforted and encouraged me.”* (Uncle Kat).

*“In the past, I had no money to spend on my family. I used to withdraw money from my employer. The employer was so kind to help every time.”* (Aunt Fang).

### 3.2. Quantitative Phase

The researchers developed a resilience scale with 39 items from the results of qualitative research and the concept of resilience [36]. Then, three experts tested the agreement of the operational definition of the items. The scale with 26 items passed the criteria and was tested on a sample of 30 elderly people with chronic diseases and depression. The researcher analysed the item-total correlation. The scale with 21 items met the criteria. The researchers then calculated the reliability of the resilience scale using Cronbach's alpha coefficient, which was 0.878, and the reliability of the 3 components of the resilience: (1) My Characteristics = 0.866, (2) My Abilities = 0.725, (3) My Dependencies = 0.959.

The results of the Pearson Product Moment correlation coefficient analysis between 21 observable variables in a sample of 310 individuals showed a correlation between 0.210 and 0.606 (*p* < 0.05). Bartlett's test for sphericity of all variables revealed no identity matrix (*χ*^2^ = 3181.076, df = 210, *p* ≤ 0.01) and Kaiser-Meyer-Olkin (KMO) value of 0.955, indicating that the data were correlated and suitable for use in confirmatory factor analysis [37].

**Figure 2:**
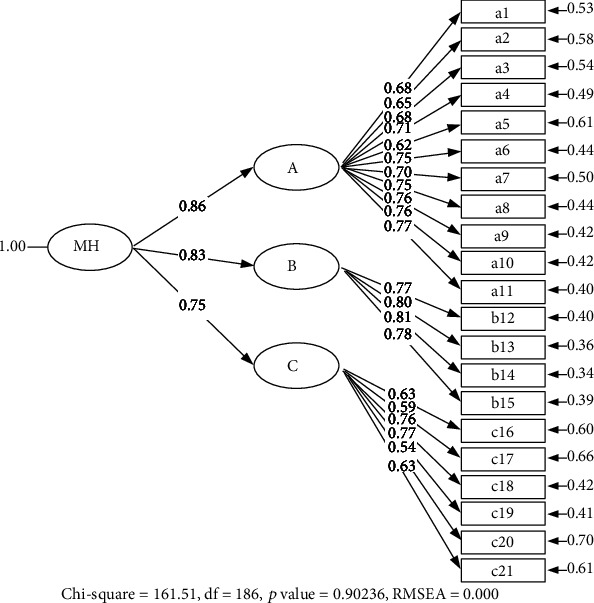
The second-order CFA of resilience scale.

The second-order CFA analysis (Figure 2) showed that the resilience model (MH), which consists of three main components: My Characteristics (A), My Abilities (B), and My Dependencies (C), was consistent with the empirical data based on the goodness-of-fit indices (chi − square = 161.51, df = 186, *p* value = 0.90, RMSEA = 0.000, SRMR = 0.02, NNFI = 1.00, CFI = 1.00, GFI = 0.95).

## 4. Discussion

This study drew on Grotberg's concept of resilience, which consists of three components: I am, I can, and I have [36], along with findings on the characteristics of resilience in elderly people with chronic diseases and depression. The qualitative results were used to develop questions and test them in the field research. The results showed the definition of resilience in elderly people in Thailand with chronic diseases and depression. It was about the internal and external attributes of elderly people who are able to maintain interpersonal and social skills in crisis situations. Thus, the resilience of elderly people with chronic diseases and depression in Thailand was composed of three components: My Characteristics, My Abilities, and My Dependencies. This was consistent with Grotberg's concept and the resilience concept of the Department of Mental Health, Thailand. When comparing the differences between the Thai Resilience Quotient (RQ-20) and the resilience scale created by the researchers, it was found that the three resilience components differed in content. The RQ-20 was used to assess individual abilities in three domains: emotional endurance, problem management, and morale [28], while the researcher-created resilience scale was based on qualitative outcomes. It was a measure of internal attributes: My Characteristics, My Abilities, and external attributes: My Dependencies. Both internal and external attributes were found to influence resilience in Thai elderly with chronic diseases and depression. In this context, the new knowledge was the content of the subcomponents in each of the main components of resilience, which was the context of Thai elderly with chronic diseases and depression. It was also found that Thai elderly with chronic diseases and depression that had high resilience would have high patience, problem-solving ability, and mental and social dependence, especially rural Thai elderly who depend on their grandchildren to take care of them. These would promote the well-being and quality of life of the elderly in Thailand [38], which is different from the elderly abroad who have the potential to be self-care and self-reliant [39].

Therefore, the researchers developed a resilience scale suitable for the context of Thai elderly with chronic diseases and depression. Quantitative tests of the developed scale showed that it was both content and structurally valid and reliable and had high psychometric properties. Health-care workers could apply this scale to elderly people with chronic diseases and depression in other regions of Thailand, although due to the limited sample size of elderly people with chronic diseases and depression, the observed variables were generated from qualitative results from a small group of participants and were less common. However, one advantage of the resilience scale was that it provided health-care workers with access to more comprehensive knowledge about resilience in elderly people with chronic diseases and depression. This enabled elderly people to be cared for according to their individual needs.

## 5. Conclusions

The resilience scale was developed to respond to the lack of a resilience scale in the context of elderly people in Thailand suffering from chronic diseases and depression and to improve health-care workers' understanding of the attributes of resilience in Thai elderly with chronic diseases and depression. The results revealed three components of resilience in the elderly: My Characteristics, My Abilities, and My Dependencies. The resilience scale was developed as a reliable and practical tool. Therefore, the health-care workers can use the components of resilience to assess the elderly and develop a resilience-promoting program specifically for elderly people with chronic diseases and depression in Thailand, as each question is based on evidence in the context of Thai elderly with chronic diseases and depression. This will be useful in caring for elderly people as it covers physical, mental, social, and spiritual aspects to improve the well-being of elderly people.

## Data Availability

All the data included in the manuscript can be accessed from the corresponding author Nanchatsan Sakunpong upon request through email (nanchatsans@gmail.com).

## References

[1] Kashaniyan F., Khodabakhshi K. A. (2015). Effectiveness of positive psychology group interventions on meaning of life and life satisfaction among older adults. *Elderly Health Journal*.

[2] Karuncharernpanit S., Limrat W., Makaroon W. (2016). Factors related to depression among older people living in homes for the aged of the Western part of Thailand. *Asian Journal for Public Opinion Research*.

[3] Steptoe A., Deaton A., Stone A. A. (2015). Subjective wellbeing, health, and ageing. *The Lancet*.

[4] David Klonsky E., May A. M., Saffer B. Y. (2016). Suicide, suicide attempts, and suicidal ideation. *Annual Review of Clinical Psychology*.

[5] World Health Organization (2021). *Noncommunicable diseases*.

[6] Li H., Ge S., Greene B., Dunbar-Jacob J. (2019). Depression in the context of chronic diseases in the United States and China. *International journal of nursing sciences*.

[7] Poole L., Steptoe A. (2018). Depressive symptoms predict incident chronic disease burden 10 years later: findings from the English longitudinal study of ageing (ELSA). *Journal of Psychosomatic Research*.

[8] Verma M., Grover S., Tripathy J. P. (2019). Co-existing non-communicable diseases and mental illnesses amongst the elderly in Punjab, India. *European endocrinology*.

[9] Gerino E., Rollè L., Sechi C., Brustia P. (2017). Loneliness, resilience, mental health, and quality of life in old age: a structural equation model. *Frontiers in Psychology*.

[10] MacLeod S., Musich S., Hawkins K., Alsgaard K., Wicker E. R. (2016). The impact of resilience among older adults. *Geriatric Nursing*.

[11] Maneerat S., Isaramalai S., Jantiya S. (2018). A guide to promote elderly resilience: a perspective from Thai context. *Asian Journal of Humanities and Social Studies*.

[12] Hassani P., Izadi-Avanji F. S., Rakhshan M., Majd H. A. (2017). A phenomenological study on resilience of the elderly suffering from chronic disease: a qualitative study. *Psychology Research and Behavior Management*.

[13] Smith J. L., Hanni A. A. (2019). Effects of a savoring intervention on resilience and well-being of older adults. *Journal of Applied Gerontology*.

[14] Huisman M., Klokgieters S. S., Beekman A. T. F. (2017). Successful ageing, depression and resilience research; a call for a priori approaches to investigations of resilience. *Epidemiology and Psychiatric Sciences*.

[15] Lima G. S., Souza I. M. O., Storti L. B., Silva M. M. . J., Kusumota L., Marques S. (2019). Resilience, quality of life and symptoms of depression among elderlies receiving outpatient care. *Revista Latino-Americana de Enfermagem*.

[16] Goins R. T., Gregg J. J., Fiske A. (2013). Psychometric properties of the Connor-Davidson resilience scale with older American Indians: the native elder care study. *Research on Aging*.

[17] Akatsuka E., Tadaka E. (2021). Development of a resilience scale for oldest-old age (RSO). *BMC Geriatrics*.

[18] Tourunen A., Siltanen S., Saajanaho M., Koivunen K., Kokko K., Rantanen T. (2021). Psychometric properties of the 10-item Connor–Davidson resilience scale among Finnish older adults. *Aging & Mental Health*.

[19] Toloie-Eshlaghy A., Chitsaz S., Karimian L., Charkhchi R. (2011). A classification of qualitative research methods. *Research Journal of International Studies*.

[20] Creswell J. W. (2014). *Research Design: Qualitative, Quantitative, and Mixed Methods Approach, fourth ed*.

[21] Guest G., Namey E., Taylor J., Eley N., McKenna K. (2017). Comparing focus groups and individual interviews: findings from a randomized study. *International Journal of Social Research Methodology*.

[22] Sakunpong N., Choochom O., Taephant N. (2016). Development of a resilience scale for Thai substance-dependent women: a mixed methods approach. *Asian Journal of Psychiatry*.

[23] Mayston R., Habtamu K., Medhin G. (2017). Developing a measure of mental health service satisfaction for use in low income countries: a mixed methods study. *BMC Health Services Research*.

[24] Rahmani A., Nithyanantham V., Fallahi A., Allahqoli L., Sadeghi N. (2019). Development and psychometric assessment of the sexual health education necessity scale: an exploratory mixed method study. *Medical Journal of the Islamic Republic of Iran*.

[25] Shirazikhah M., Mirabzadeh A., Sajadi H. (2019). Development and psychometric properties of the physical rehabilitation services acceptability questionnaire. *Medical Journal of the Islamic Republic of Iran*.

[26] Institute of Geriatric Medicine (1999). *Mini-Mental State Examination Thai-Version 2002*.

[27] Kongsuk T., Arunpongpaisal S., Janthong S., Prukkanone B., Sukhawaha S., Leejongpermpoon J. (2018). Criterion-related validity of the 9 questions depression rating scale revised for Thai central dialect. *Journal of the Psychiatric Association of Thailand*.

[28] Intasitti S., Chulkeeree S. (2020). *Change Bad to Good Mental Health Power RQ: Resilience*.

[29] Vella S. L. C., Pai N. B. (2019). A theoretical review of psychological resilience: defining resilience and resilience research over the decades. *Archives of Medicine and Health Sciences*.

[30] Morrow S. L. (2005). Quality and trustworthiness in qualitative research in counseling psychology. *Journal of Counseling Psychology*.

[31] Joshi A., Kale S., Chandel S., Pal D. K. (2015). Likert scale: explored and explained. *British Journal of Applied Science & Technology*.

[32] Turner R. C., Mulvenon S. W., Thomas S. P., Balkin R. S. (2002). *Computing Indices of Item Congruence for Test Development Validity Assessments, in 27th Annual SAS Users’ Group International Conference*.

[33] Kline P. (2015). *A Handbook of Test Construction (Psychology Revivals): Introduction to Psychometric Design*.

[34] Cronbach L. J. (1951). Coefficient alpha and the internal structure of tests. *Psychometrika*.

[35] Morata-Ramírez M. D. L. Á., Holgado-Tello F. P. (2012). Construct validity of Likert scales through confirmatory factor analysis: a simulation study comparing different methods of estimation based on Pearson and polychoric correlations. *International Journal of Social Science Studies*.

[36] Grotberg E. H. (1995). *The International Resilience Project: Research and Application*.

[37] Kim S., Sturman E., Kim E. S. (2015). *Structural Equation Modeling: Principles, Processes, and Practices, in the Palgrave Handbook of Research Design in Business and Management*.

[38] Juthavantana J., Sakunpong N., Prasertsin U., Charupheng M., Lau S. H. (2021). An integrative counselling program to promote active ageing for older people in Thai nursing homes: an intervention mixed methods design. *BMC psychology*.

[39] Foster L., Walker A. (2021). Active ageing across the life course: towards a comprehensive approach to prevention. *BioMed Research International*.

